# Examining Factors in the Research Institute on Addictions Self-Inventory (RIASI): Associations with Alcohol Use and Problems at Assessment and Follow-Up

**DOI:** 10.3390/ijerph6112898

**Published:** 2009-11-24

**Authors:** Robert E. Mann, Gina Stoduto, Rosely Flam Zalcman, Thomas H. Nochajski, Louise Hall, Patricia Dill, Elisabeth Wells-Parker

**Affiliations:** 1 Social, Prevention and Health Policy Research, Centre for Addiction and Mental Health, 33 Russell Street, Toronto, ON, M5S 2S1, Canada; E-Mails: gina_stoduto@camh.net (G.S.); rosely_flam@camh.net (R.F.Z.); 2 Dalla Lana School of Public Health, University of Toronto, Toronto, ON, Canada; 3 School of Social Work, University at Buffalo SUNY, 660 Baldy Hall, Amherst, NY 14260, USA; E-Mail: thn@buffalo.edu; 4 Social Science Research Centre, Mississippi State University, Mississippi State, MS 39762, USA; E-Mail: bwparker@ssrc.msstate.edu

**Keywords:** convicted impaired drivers, assessment, screening instrument, factor analysis

## Abstract

Impaired driving is a leading cause of alcohol-related deaths and injuries. Rehabilitation or remedial programs, involving assessment and screening of convicted impaired drivers to determine problem severity and appropriate programs, are an important component of society’s response to this problem. Ontario’s remedial program, Back on Track (BOT), involves an assessment process that includes administration of the Research Institute on Addictions Self-Inventory (RIASI) to determine assignment to an education or treatment program. The purpose of this study is to identify factors within the RIASI and examine how factor scores are associated with alcohol use and problem indicators at assessment and six-month follow-up. The sample included 22,298 individuals who completed BOT from 2000 to 2005. Principal component factor analysis with varimax rotation was conducted on RIASI data and an eight factor solution was retained: (1) Negative Affect, (2) Sensation Seeking, (3) Alcohol-Quantity, (4) Social Conformity, (5) High Risk Lifestyle, (6) Alcohol Problems, (7) Interpersonal Competence, and (8) Family History. Regression analyses were conducted to examine associations between factors and alcohol and problem measures obtained at assessment and at follow-up. Most factors, except for Interpersonal Competence, were associated with more alcohol use and problems at assessment. A similar pattern was observed at 6-month follow-up, but interestingly some factors (Negative Affect, Sensation Seeking, Alcohol-Quantity and Family History) predicted fewer days of alcohol use. The Interpersonal Competence factor was associated with significantly lower levels of alcohol use and problems at both assessment and follow-up. This work suggests that the RIASI provides information on several domains that have important relationships with alcohol problem severity and outcomes.

## 1. Introduction

Driving while impaired by alcohol (DWI) continues to be a major cause of alcohol-related injuries and deaths in Canada and elsewhere. Remedial programs for convicted drinking drivers have been implemented in most jurisdictions in North America over the past few decades. Research has shown that such programs can have a positive influence on the beliefs and attitudes held by convicted drivers as well as decreasing levels of recidivism, collisions and improving the overall health status of DWI offenders [1–4]. The goal of remedial programs is to reduce the likelihood that participants will drive after drinking in the future, which will lead to a reduction in the number of alcohol-related collisions [3]. Effective remedial programs should influence these statistics, since repeat offenders are over-represented in collisions; about one out of eight intoxicated drivers involved in fatal crashes have had a previous DWI conviction within three years prior to the crash [5]. Therefore, remedial programs also provide a preventative measure by identifying DWI offenders who may be at risk of re-offending as well as an opportunity to direct treatment interventions to target this population [6].

The use of screening and assessment instruments to identify the most appropriate program options is an important component of remedial programs for convicted drinking drivers [1,7]. By identifying offenders suited for different types of interventions the outcomes of remedial programs can be enhanced [8]. Researchers have looked for other variables that may effectively identify offenders at risk for continued problem alcohol use and recidivism in order to better understand the aetiology of these problems, and aid in treatment matching and prevention [9]. By identifying significant predictors of problem substance use and DWI recidivism screening can seek to identify individuals at high risk for continued problems [6].

Many studies indicate that the DWI population is a heterogeneous group, differing on demographic, behavioral, psychological and social measures [10]. Schell *et al.* [11] found that self-reported drinking driving was predicted by frequent drinking, positive expectancies about alcohol and low levels of socially desirable response bias. It has been found that repeat drinking drivers tend to score higher than non repeat offenders on sensation seeking, hostility, depression and psychopathic deviance and lower on assertiveness and emotional adjustment [12]. Psychiatric disorders commonly co-occur with alcohol disorders and this is an important factor for outcome of remedial programs as they do not usually address both substance abuse and psychiatric disorders [13]. Wells-Parker and Williams [14] found that DWI offenders who reported higher levels of depressed mood were more likely than those with lower levels to receive a subsequent drinking driving conviction. Nochajski *et al.* [15] found that drinking drivers who re-offended within a 12-month period of their first DWI offence were characterized by sensation seeking, impulsivity and general deviance. Age, gender, marital status, and race/ethnicity have also been found to differentiate DWI recidivists. In general males are much more likely to re-offend than females [13]. Interestingly, while cross-sectional studies show repeat offenders to be older than first time offenders longitudinal studies indicate that individuals under the age of 30 are more like to continue drinking and driving (e.g., [16]). A 12-year follow-up study [17] found that a poor driving record prior to and following an initial DWI was predictive of recidivism, suggesting that DWI is often one of a cluster of problem behaviors which may include drug use, traffic violations and financial or occupational irresponsibility. This may be a result of a poor decision-making lifestyle rather than just alcohol abuse, which if true may also suggest program innovations to improve outcome. If DWI offenders are found to have individual characteristics or co-morbid conditions associated with continued problematic substance use and recidivism, the success of remedial programs may be improved if these factors are taken into account.

Due to such diversity within the population research is beginning to focus on identifying subsets of DWI offenders in an effort to further guide research and inform clinical practice [1]. Thus research has shifted to development of screening instruments which measure a number of other dimensions such as personality, emotional and motivational factors, lifestyles, cognitive factors and psychiatric problems that can often co-exist with alcohol abuse and dependence [18]. Some instruments include disguised or indirect indicators of substance use and related problems. One screening instrument, the Research Institute on Addiction Self-Inventory (RIASI), was designed specifically for use with convicted drinking drivers [19,20]. Initially validated on samples in New York State, the reliability and validity of the instrument has been confirmed in studies from Ontario [21]. The RIASI developers purposely included items reflecting a variety of domains affected by, leading to, or associated with, alcohol problems [19,22].

**Figure 1.**
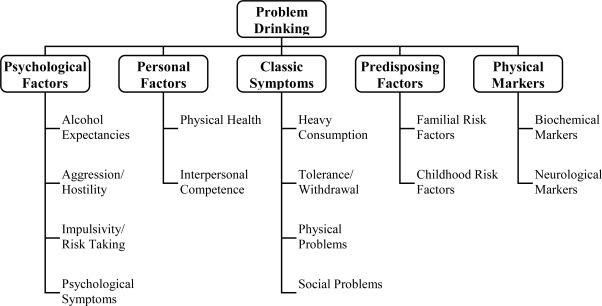
Content domains sampled in construction of the RIASI.

The RIASI is a 52 item instrument that measures distal (hostility/aggression, sensation seeking, depression, anxiety, interpersonal competence, childhood risk factors, social problems such as criminal history, health issues) and proximal factors (current drinking habits, preoccupation with alcohol, alcohol beliefs, use of alcohol to alleviate problems, and family history) associated with alcohol or drug problems (see Figure 1). The instrument’s developers originally suggested that a total of any 10 positive responses would require the individual to go for the more intense clinical evaluation [19].

The samples used for development of the RIASI were large enough to allow for examination of subgroups based on age, race, gender and region of New York state. Information concerning internal consistency shows that the magnitude of the Cronbach’s alpha coefficient remained relatively stable across different samples of convicted drinking drivers; 0.819 for a 1992 New York statewide sample of 5,059 participants; 0.819 for a 1993 sample of 1,024 participants from Erie County in Western New York; 0.807 for a 1993 sample of 209 convicted drinking-drivers from New Jersey; 0.814 for a 1994 sample of 1,477 DDP participants from New York State; and 0.808 for a 1994 sample of 100 high risk convicted drinking-driver offenders from Erie County; 0.893 found for a sample of 113 individuals from an Onondaga county (for more information on reliability and validity see [19]). The validity of RIASI has also been confirmed in the convicted drinking driver population in Ontario [21]. In addition to a total score based on all the items on the instrument, Nochajski and colleagues developed a recidivism subscale of 15 items on the instrument that was able to correctly identify over 80% of individuals who were rearrested for drinking driving over a two-year period [20,22]. Recommended cutoffs for referral of participants to more extensive follow-up were 9 on the total score, and 3 on the recidivism scale [22].

The RIASI has proven to be a valuable instrument for screening convicted drinking drivers in New York, Ontario, and other jurisdictions [e.g., 21,22]. It is also possible that the method of construction of the instrument, by including both proximal and distal indicators of alcohol problems such as depression, sensation seeking, and social problems such as criminal history, could provide additional information that would be valuable for program assignment purposes. The aim of this study is to identify factors within the RIASI and explore the validity of these factors by examining how they are associated with alcohol use and problem indicators at assessment and 6-month follow-up based on data from a large sample of convicted impaired drivers in a remedial program in Ontario.

## 2. Methods

Convicted impaired drivers in Ontario are required to complete the Back on Track (BOT) remedial measures program following a mandatory period of driver’s license suspension, before they can get their license reinstated. The assessment is the first step in completion of BOT and consists of the Research Institute on Addiction Self-Inventory (RIASI) [19,22], Alcohol Dependence Scale (ADS) [23] and Drug Abuse Screening Test (DAST) [24]. The ADS and DAST are widely used among professionals working with clients seeking treatment for substance abuse. Assignment to the education or treatment program is based on a threshold score being reached on any of three instruments administered at assessment, the RIASI, ADS and DAST. The threshold scores used for assignment to the treatment program are as follows: ADS ≥ 14, DAST ≥ 6, RIASI Total (RIASI-T) score ≥ 9 and RIASI Recidivism (RIASI-R) score ≥ 6. The threshold for RIASI-R scores in Ontario’s program was increased from that recommended by Nochajski *et al*. [22] because all participants in Ontario’s program were receiving a remedial intervention, and the instrument was used to identify those who would receive a longer, more intensive program rather than a follow-up screening. Other measures are also collected at both assessment and follow-up including the Adverse Consequences of Substance Use Scale (ACSUS) [25], measures of substance use, and contacts with health services in the 90 days prior to the contact (assessment or outpatient drug/alcohol treatment facility sessions, mental health centre or facility sessions as an outpatient, employee assistance program sessions, family and or marital counseling services, private doctors office visits, self-help meetings for alcohol/drug problem and self-help meetings for issues other than alcohol/drug problems).

Assessment interviews are conducted at 29 sites across the province by trained addictions professionals using a computerized (web-based) protocol. Most assessment interviews (88.5%) were performed in person, with the remainder by telephone, and on average were 60 minutes long. The follow-up interview was performed 6 months after successful completion of the education/treatment program (and required 30 minutes to complete). The education program aims to educate participants about the effect of alcohol and other drugs on driving performance; behavioral effects of alcohol intake; consequences of impaired driving; their own attitudes, beliefs and behaviors in relation to impaired driving; ways to avoid situations that involve alcohol, other drugs and driving; and making plans to avoid another impaired driving offence. The primary difference between the education and the treatment program is the addition of activities designed to enhance treatment program participants’ coping skills in the following areas: dealing with stress; communicating assertively; improving leisure time; and managing anger.

We employed exploratory factor analysis, using principal component analysis [26] to extract factors that account for correlations between RIASI items. The Kaiser criterion was used to retain only factors with eigenvalues > 1. The Scree test [27] showed that eigenvalues dropped considerably from the first to the second factor, less so from second to third, and stayed at the same level from the ninth factor onward. Therefore, an eight factor solution was retained. Using varimax rotation, we obtained a simple structure solution while maintaining orthogonal factor axes. Rotated factor loadings over 0.6 are considered large and moderately large if higher than 0.3.

Data were analyzed using the STATA statistical software package. Concurrent validity of the factors was assessed by regression analysis, where measures such as ADS, DAST and previous drinking driving convictions were regressed onto the factor scores while controlling for age and gender. The predictive validity of the factors was assessed by regression analyses, where alcohol use in the 90 days preceding follow-up, consequences of substance use experienced in the 90 days preceding follow-up, and contacts with health services (including addictions programs) in the follow-up period were regressed onto factor scores while controlling for age and gender. T-tests were used for accepting or rejecting the null hypothesis related to the regression parameters. We set the alpha level for identifying significant regression coefficients at 0.005.

## 3. Results

### 3.1. Sample Characteristics

Table 1 presents data on sample demographic characteristics. The sample consists of 22,298 individuals who completed all components of the BOT by March 31, 2005. The clients completed their assessments beginning November 1, 1999. The majority of clients (about 96%) completed the follow-up within seven months of participating in their assigned 8-hour education or 16-hour treatment program. The sample was predominantly male (88%), average age 46 years, average 13 years of education, modal income $20,000–$49,999, 44.4% married and 35.8% single, and 72.1% employed. The mean scores on the RIASI-T, RIASI-R, ADS and DAST were 6.8, 3.3, 1.8, and 0.3 respectively. One quarter (25.8%) reported that they had a previous DWI offence. A total of 16,450 (73.8%) were assigned to the education program and 5,848 (26.2%) to the treatment program.

### 3.2. Identification of Factors

RIASI items loading on factors are shown in the Appendix. The proportion of variance explained by each factor corresponds to the eigenvalue and equals the sum of the squares of the factor loadings. The first component had the highest eigenvalue (5.70). The aim is to account for the maximum amount of variance with the smallest number of components. An 8-factor solution explained 31% of the total variance. The 8-factor solution retained showed substantial correspondence with the conceptually-defined items originally included by Nochajski *et al.* [19] (see Figure 1). The first factor included five items reflecting depressed mood, three reflecting anxiety, two reflecting hostility, and one reflecting sensation seeking. Since this factor seemed to reflect negative affect more generally, rather than depressed mood specifically, it was labeled Negative Affect. The second factor extracted loaded highly on items reflecting seeking or undertaking risky things for fun, or tendencies towards more impulsive behavior, therefore, labeled Sensation Seeking. The third factor items mainly reflected higher quantities of alcohol consumed on drinking occasions (largest number of drinks consumed in a 24-hour period, how many drinks usually consumed on a drinking occasion), other indicators of heavier drinking (more drinking days per week, money spent on alcohol per week), and one item suggesting higher tolerance for alcohol (how many drinks before feeling the effects). This factor was labeled Alcohol-Quantity. The fourth factor had four items; three came from a pilot lie scale [19], which identified behaviors that reflect a response pattern of endorsing the socially desirable response, even though it would be an unlikely response. For example, item 51 states, ‘I sometimes feel resentful when I don’t get my way.’ A response of ‘no’ on this item is considered to reflect a desire to provide a socially appropriate response, as opposed to an honest or accurate response. One item that was originally included to reflect hostility also loaded on this factor. This factor was labeled Social Conformity. The fifth factor items reflected criminal activities, high risk driving, and a history of being injured, which reflect a willingness to take risks or disregard societal rules, therefore labeled High Risk Lifestyle. The sixth factor labeled Alcohol Problems included items on symptoms of alcohol dependence or loss of control (e.g., I hardly ever drink more than I plan to), and acute and chronic consequences of excessive drinking (e.g., I was referred for a liver test, or a blood test for liver enzymes), and one item reflecting depression, which is often associated with excessive drinking. Higher scores on this factor reflected lower alcohol problem levels. The seventh factor labeled Interpersonal Competence appeared to reflect willingness and ability to act based on one’s own judgment. Items in this factor suggested assertiveness, internal locus of control, and an assertive attitude. The eighth factor extracted included items that reflected alcohol-related problems in family members, and was therefore labeled Family History. As with the Alcohol Problems factor, higher scores on the Family History factor reflected lower likelihood of being positive on family history measures.

### 3.3. Association between RIASI Factors and Assessment Measures

Table 2 presents data on the association between the RIASI factors and measures of alcohol use and problems for the 90 days preceding the assessment. The Negative Affect, Alcohol-Quantity, Social Conformity, High Risk Lifestyle, and Alcohol Problems factors were associated with more days of drinking alcohol and more drinks per drinking occasion. Higher levels of Sensation Seeking and Family History were associated with more drinks per drinking occasion. Higher scores on the Interpersonal Competence factor predicted significantly fewer days using alcohol, as well as fewer drinks per occasion.

Higher levels of Negative Affect, Alcohol-Quantity, Social Conformity, High Risk Lifestyle, Alcohol Problems and Family History factors were associated with more DWI charges and convictions, and higher scores on the ADS and DAST. Higher levels of Sensation Seeking were found to be positively associated with the ADS and DAST, but associated with fewer previous DWI convictions. A higher score on the Interpersonal Competence factor was associated with fewer DWI charges and convictions, and lower scores on the ADS and DAST.

### 3.4. Association between RIASI Factors and Follow-Up Measures

Table 3 presents data on the associations between the RIASI factors and measures obtained for the 90 days preceding the follow-up interview. Higher levels of Negative Affect are associated with significantly fewer drinking days at follow-up but not related to number of drinks per occasion. The Sensation Seeking factor was associated with significantly fewer drinking days but also was associated with more drinks per occasion. Higher scores on Alcohol-Quantity and on Social Conformity predicted more days using alcohol and more drinks per drinking occasion. Higher scores on the High Risk Lifestyle factor predicted more drinks per drinking occasion. More Alcohol Problems predicted fewer days using alcohol, while a greater Family History involvement predicted more drinks per drinking occasion. Finally, higher scores on the Social Competence factor predicted fewer drinks per drinking occasion.

The relationships between the RIASI factors and consequences of substance use from the follow-up ACSUS [25] are also presented in Table 3. Higher levels of Negative Affect and more Alcohol Problems predicted more adverse consequences in every area: health, memory, mood, relationships, aggression, school or work, legal and financial. Similar consistent relationships with all consequence measures except for school/work problems were observed for the Alcohol-Quantity factor. The Sensation Seeking factor was negatively related to school /work problems, indicating that higher scores on this factor were associated with fewer problems in this domain, but higher scores in all others.

The Social Conformity factors showed no significant relationship with memory, aggression, school/work, legal and financial problems, but a significant positive relationship with all others. More Family History of alcohol-related problems showed no significant association with relationship, aggression, and school/work problems, but it showed a significant association with all other problems. Finally, higher scores on the Interpersonal Competence factor predicted significantly fewer adverse consequences in all domains, with the exception of school or work problems.

Table 4 presents the associations between the RIASI factors and use of health services in the follow-up period. Higher levels of the Negative Affect factor predicted more use of all types of health services except for self-help programs for other than alcohol or drug problems. More Alcohol Problems similarly predicted higher use of all services. Higher levels of Sensation Seeking predicted more contact with Employee Assistance programs and self-help meetings for alcohol/drug problems. Higher scores on the Alcohol-Quantity factor predicted more contacts with drug/alcohol treatment facilities, Employee Assistance programs, private physicians, and self-help meetings for alcohol or drug problems. Respondents with higher Social Conformity scale scores had more contacts with drug/alcohol treatment facilities, outpatient mental health facilities, and self-help meetings for alcohol or drug problems. Higher scores on the High Risk Lifestyle factor predicted more contacts with self help programs for alcohol or drug problems. Greater Family History of alcohol problems was related to more contacts with drug/alcohol treatment facilities, self help for alcohol/drug problems and self-help for other problems. Higher scores on High Risk Lifestyle and lower scores on the Interpersonal Competence factors predicted more contacts with drug/alcohol treatment and self-help meetings for alcohol/drug problems. Scores on the Sensation Seeking factor were unrelated to any measures of service use at follow-up.

## 4. Discussion

Remedial programs are an effective part of efforts to prevent impaired driving, and screening offenders to determine problem severity and appropriate programs is a recommended component of these programs [1]. Ontario’s Back on Track remedial program assessment includes administration of the RIASI [19] to determine if participants should complete the education or treatment program. The RIASI includes items reflecting domains known to be strongly related to alcohol use and problems but which are not indicators of alcohol use and problems as such. However, to date no studies have examined if people’s responses reflect these domains, and similarly if meaningful groups of individuals can be differentiated based on responses on the RIASI. In this study we have identified factors on the RIASI that are closely related to the original domains sampled in the development of the instrument. As well, the concurrent and predictive validity of these factors is supported by the findings that they show important relationships to alcohol use and problems at assessment and at six month follow-up.

An eight factor solution appeared to best represent the data: (1) Negative Affect, (2) Sensation Seeking, (3) Alcohol-Quantity, (4) Social Conformity, (5) High Risk Lifestyle, (6) Alcohol Problems, (7) Interpersonal Competence, and (8) Family History. The first factor seemed to reflect depressed mood and hostility; the second loaded highly on items reflecting seeking or undertaking risky things for enjoyment. The Alcohol-Quantity factor reflected higher quantities of alcohol consumed on drinking occasions and other indicators of heavier drinking. The fourth factor included items that reflect a response pattern of endorsing the socially desirable response. The High Risk Lifestyle factor included items that reflected involvement in criminal activities and a history of being injured. The Alcohol Problems factor included items that reflected symptoms of alcohol dependence or loss of control, but interestingly was negatively related to problem indicators such that higher scores on the factor reflected lower problem indicators as reflected on the factor loadings. The seventh factor appeared to reflect assertiveness and internal locus of control. The eighth factor included items that reflected alcohol-related problems in family members, and it, like the Alcohol Problems factor, was negatively related to to family history indicators such that higher scores on the factor reflected lower family history indicators. In general, these factors correspond well, although not perfectly, with the conceptual domains that formed the basis for item selection for the RIASI. Three factors reflect specific alcohol factors, with five reflecting domains that do not have an obvious connection to alcohol use. Interestingly, the factor that appeared to account for the largest proportion of the variance in the scores on RIASI was the Negative Affect factor, a non-obvious indicator. This points to the value of non-obvious indicators and suggests that more attention be given to understanding their significance in the population of drinking drivers.

All of the factors except for Interpersonal Competence, Alcohol Problems and Family History showed a positive impact on assessment measures, such that higher levels of these factors were associated with more alcohol use and problems at assessment. Higher levels of Interpersonal Competence were associated with less alcohol use and fewer problems at assessment, and while there were negative relationships between the Alcohol Problem and Family History factors and alcohol use and problems this is explained because higher scores on these factors reflect fewer problems and less of a family history of alcohol problems. A similar pattern was observed at follow-up, with some interesting and important exceptions. Higher levels of Negative Affect and Alcohol Problems predicted fewer drinking days in the 6-month follow-up interview, which supports recent suggestions that higher levels of indicators like Negative Affect at the beginning of a treatment intervention could be a positive prognostic indicator under some circumstances [28,29]. These results suggest that responses on the RIASI are, first of all, valid indicators of alcohol use and related problems. They also suggest that the non-obvious indicators are valid indicators and predictors of alcohol use and problems, and thus provide additional support for the use of this instrument.

When screening instruments for drinking drivers are used typically only one or two scores contribute to programming and clinical decisions. However, this work has demonstrated that the RIASI provides information on several domains, all of which appear to be related to alcohol problem severity and outcomes. Subgroups of convicted drinking drivers appear to exist in the drinking driver population differentiated by the factors uncovered in the RIASI, and these subgroups may respond differently to interventions.

### 4.1. Negative Affect

The observations on the Negative Affect factor are consistent with a growing body of literature on the importance of negative affect for understanding drinking drivers (e.g., [29]), and recently investigators have begun to consider the importance of this factor for remedial and rehabilitative efforts [28–31]. It is interesting to note here that this factor included items reflecting depressed mood, but also other states including anxiety, hostility and sensation seeking. Thus, we chose to describe this factor as Negative Affect, rather than depression, to reflect a more general negative state. Since the psychometric properties of instruments measuring depression are also complex [32], it would be interesting to determine how this state corresponded to more clinically-defined depression. One possibility is that Negative Affect observed here is importantly related to these individuals’ recent experiences with the legal system and with the social, economic and personal consequences of that experience. Thus the implications of this Negative Affect state may be different than the implications of clinical depression. Certainly the observation that higher levels of this state reflected more alcohol use prior to program entry, and less alcohol use at follow-up, suggests that this state should not be interpreted simply as in indicator of more serious problems and worse outcome. One interesting possibility is that because the main purpose of the BOT program is to reduce driving after drinking, elevated levels of the Negative Affect factor reflected a motivational state that somehow was conducive to achieving the specific goals of the program [29]. These results may be consistent with those of Wells-Parker and Williams [14], who observed that individuals high on the depression subscale of the Mortimer-Filkins measure were also more responsive to brief individual interventions to a program for convicted drinking drivers. Additionally, it appeared that higher levels of the Negative Affect factor also predicted more utilization of addictions and health care services at follow-up, which also might suggest that these individuals were more motivated to take additional steps to deal with personal problems. The results of this work provide important support for the potential value of understanding negative affect and its implications for convicted drinking drivers [29], and also points to the potential value of using subscores of the RIASI for program assignment purposes.

### 4.2. Sensation Seeking

Sensation seeking as a concept [33] involves a propensity to seek novel and intense sensations and experiences, including a willingness to take risks to attain those experiences. Sensation seeking is reflective of higher likelihood for risky driving, including impaired driving [34,35]. Higher levels of sensation seeking are associated with poorer outcomes in alcohol and drug treatment, including reduced likelihood of treatment completion [36]. At assessment, the Sensation Seeking factor showed an interesting pattern of associations with use of the various substances. The relationship with alcohol use measures was complex, with Sensation Seeking being associated with fewer drinking days, but more drinks per occasion. A strong pattern of association with consequences of alcohol use was seen. Sensation Seeking was positively associated with all the adverse consequences of substance use, with the exception of a negative relationship with school and work problems. Interestingly, Sensation Seeking predicted higher scores on the ADS and DAST, but fewer previous drinking driving charges or convictions. Individuals who scored higher on Sensation Seeking were less likely to report use of alcohol at follow-up. Interestingly, these relationships did not translate into increased health care utilization. This could reflect a tendency for individuals with higher levels of Sensation Seeking to avoid health care services.

### 4.3. Alcohol-Quantity

Consistent with previous studies [10], we observed that higher scores on the Alcohol-Quantity factor predicted more use of alcohol, heavier drinking, and more DWI arrests and convictions at assessment and more adverse consequences of substance use and greater use of health services at follow-up. This suggests that elevated scores on the Alcohol-Quantity factor is a clear indicator of more problems at both assessment and follow-up. A large number of studies attest to the importance of quantity of alcohol consumed on a drinking occasion or over the course of a period of time, such as a day, week or month, as a major determinant of the risk of drinking driving, collisions, and other harms [37–39]. The Alcohol-Quantity factor appeared to be one of the most robust correlates and predictors of alcohol use, problems and negative outcomes among the factors derived. In view of the drinking driver population considered here, it may not be surprising that the Alcohol-Quantity factor appears to figure prominently as a problem indicator in these analyses.

### 4.4. Social Conformity

Three of the four items that constitute the Social Conformity scale were originally included on the RIASI by Nochajski *et al.* [19] in an effort to measure purposeful efforts to distort results. The scores on the Social Conformity scale, in general, showed similar significant associations with the measures obtained at assessment, and with the measures obtained at follow-up, to most of the other scales. Higher levels of Social Conformity were significantly related to more drinks per drinking day, more days using alcohol, more previous DWI charges and convictions, and higher scores on the ADS and DAST at assessment. At follow-up, higher scores were associated with more use of alcohol, higher levels of negative consequences except for school/work problems, and more involvement with most health services. These results do indicate that scores on the Social Conformity scale are associated with and can predict alcohol and substance use and problems, even though their content is unrelated to these measures. This finding does validate the potential usefulness of this measure as an indicator of problems. However, additional work is needed to determine the particular value of this measure as a problem indicator when other factor scores are suggesting lower problem levels.

### 4.5. High Risk Lifestyle

The High Risk Lifestyle factor consists of items reflecting commission of various crimes and experience of injury. These items are also related to the presence of Anti-Social Personality Disorder (ASPD), a condition which has been found to be more common in groups of convicted drinking drivers [31]. Previous studies have shown that individuals who have more criminal involvement, or are diagnosed with ASPD at assessment, also have higher levels of alcohol use and other problem indicators [10,40]. Our results are consistent with these previous findings. We observed that higher scores on this factor predicted more drinks per drinking day, more adverse consequences of substance use, and more contacts with most forms of health services at follow-up. Thus, higher scores on this factor appear to predict increased risk of adverse outcomes in this population.

### 4.6. Alcohol Problems

The Alcohol Problems factor reflects health and social consequences of drinking and a drinking style suggesting loss of control. Experience of problems in association with loss of control over drinking are two major defining characteristics of alcohol dependence, and thus this scale may be reflecting this. Alcohol dependence is considered to be a more serious and advanced form of problem, and includes the development of health-related problems such as alcohol-related liver disease [38]. The Alcohol Problems factor was associated with more days of drinking and more drinks per drinking day, more DWI charges and convictions, and higher scores on the ADS and DAST at assessment. At follow-up, more Alcohol Problems were associated with higher levels of all adverse consequences, and more contacts with all health services. These findings are consistent with this factor being a strong indicator of increased problems and risk. On the other hand, higher scores on this factor were associated with fewer days of alcohol use, but were not predictive of number of drinks per occasion at follow-up. This pattern is the same as that observed for the Negative Affect factor, and at first glance seems counter-intuitive. However, these results may also be suggesting that high scores on this factor could reflect the individual’s recognition of their own alcohol problems, and possibly indicates a desire to address these problems. Thus, scores on this factor reflecting more alcohol problems could be reflecting both a need and a desire for more help in dealing with these alcohol problems. In interpreting this factor it is important to keep in mind that high scores on the factor reflect lower problem levels, and thus it may be worth considering reverse-scoring this factor in any practical applications.

### 4.7. Interpersonal Competence

The Interpersonal Competence factor included items that reflect self-confidence, assertiveness, and a perceived ability to make and follow plans. Higher scores on this factor were associated with fewer days of alcohol use, fewer drinks per occasion, fewer previous DWI charges and convictions and lower ADS and DAST scores at assessment. Higher scores predicted better outcomes at follow-up, including lower levels of alcohol, fewer adverse consequences of use, and fewer contacts with addiction treatment and self-help resources. Thus, this factor appears to have important value as a positive prognostic indicator. Previous studies have demonstrated the importance of concepts and factors that are related to the Interpersonal Competence factor identified here, including self-efficacy, self-confidence and assertiveness. These factors have been linked, in the context of addictions, to more positive outcomes and better responsiveness to treatment interventions [29,31,41]. The results observed here with the Interpersonal Competence factor confirm these observations and also support the validity of this factor as differentiated from other factors seen in the RIASI.

### 4.8. Family History

Individuals with a family history of alcohol problems are themselves more likely to develop alcohol problems, to develop them at a younger age, and be more likely to experience emotional problems, hyperactivity, and conduct problems [42]. These risks may be related to both environmental influences and to genetic factors [43,44]. The association between the Family History factor and measures obtained at assessment confirmed expectations that individuals with a family history of alcohol problems would demonstrate more drinks per occasion (but interestingly, not with number of drinking days), more DWI charges and convictions, and higher scores on the ADS and DAST. More extensive family involvement with alcohol also predicted more problems at follow-up, including more drinks per occasion, more negative consequences (with the exception of problems with relationships, aggression and with school/work), and more contacts with addictions services and self-help groups. This is consistent with a large body of research (e.g., [42,43]), and points to the validity of this factor in the RIASI and its potential utility as a marker for adverse outcomes. As with the Alcohol Problems factor, it is important to keep in mind that high scores reflect lower family involvement in alcohol problems, and that reverse scoring might be considered in any practical applications of this analysis.

### 4.9. Limitations

Several limitations must be kept in mind when considering these results. One limitation of these results is that they are based on self-report measures which may be subject to a variety of factors that may affect their validity. Additionally, while participants are not required to attend BOT by courts, attendance is necessary if they want their driver’s license reinstated. Thus, there may be demand characteristics in the program that may act to influence participants’ self-reports. The available evidence indicates that self-report measures of alcohol and drug use are generally reliably and valid (e.g., [45,46]). Nevertheless, under-reporting of drug use and previous legal problems in this population has been noted in the literature [47]. An additional limitation of these results is that they may be specific to the time period covered in this research, or may have been affected by factors that occurred during the time when these data were collected, although we are not aware of any policy changes or similar factors that would have affected the nature of the convicted drinking driver population over this period. Thus, while of substantial interest, these results need to be replicated and extended in further research.

Another limitation of these observations is that they do not consider relationships of the factor scores with traffic safety consequences, a key concern underlying the creation of remedial programs, and a key goal of programs for participants is to reduce drinking driving recidivism and collisions. However, it is clear that a necessary precursor to impaired driving is excessive alcohol use, and thus the measures considered here are of substantial importance to understanding how to prevent impaired driving. Nevertheless, it will be important to examine the traffic safety correlates of RIASI factor scores in future studies.

## 5. Conclusions

Considering results across factors, several observations are of interest. A general pattern observed (with important exceptions) was that factor scores reflecting higher problem levels on most factors were associated with more alcohol use at assessment and follow-up, and higher levels of alcohol related problems at assessment and follow-up. One factor, the Interpersonal Competence factor, predicted lower levels of use and fewer problems at assessment and follow-up. In considering the severity-based assignment scheme used in BOT to assign clients to the shorter education or longer treatment programs, some RIASI scales may predict severity, or higher problem levels, with more precision and thus permit more efficacious assignment processes. Further exploration of these possibilities could provide very useful information for program improvement purposes.

Some factors predicted significantly fewer drinking days at follow up, even though they predicted more alcohol using days at assessment (Negative Affect and Alcohol Problems). The specific and possibly beneficial relationship on days using alcohol at follow-up could indicate that a specific impact of being in the Back on Track program may be seen here. The program is designed to address heavy drinking as a determinant of drinking driving, and to provide clients with ways to reduce heavy drinking as well as separate drinking from driving. Thus, those for whom this information is most relevant, *i.e.*, heavy drinkers, may be making the most use of it. Similarly, higher levels of negative affect at program intake may be a potential marker of higher levels of motivation which in turn may support beneficial treatment effects [29].

There are several ways in which the identification of these factors may help improve program assignment practices. While current program assignment processes are based on total RIASI scores, or scores on the empirically-derived recidivism subscale, it is possible that scores on one or more of the factors identified here may also predict differential responsiveness to different program types. For example, previous research has shown that drinking drivers who have higher scores on a depression measure derived from the Mortimer-Filkins assessment instrument have improved results when they receive a supplemental brief individual intervention [14]. It might thus be possible that individuals who have low scores on the RIASI but elevated levels on the Negative Affect factor, who might otherwise be assigned to a briefer intervention with a larger group size, may experience an improved result if assigned to a longer intervention with a smaller group size. One way that these possibilities might be explored is through the assessment of whether or not these factor scores might moderate the effects of Back on Track’s education or treatment programs on program outcome.

It is important to keep in mind that while these analyses point to the potential value of the RIASI factor scores in refining program assignment practices, they do not yet provide an empirical basis for modifying these practices. Future research should assess if there is any added value to including factor information in program assignment decisions.

## Figures and Tables

**Table 1.** Sample characteristics at assessment. Back on Track remedial program clients (N = 22,298).

**Gender** Female	12%
Male	88%

**Age** mean (SD)	46 (12)

**Education** mean (SD)	13 (5)

**Income** < $20,000	22.5%
$20,000–$49,999	52.6%
$50,000–$79,999	16.7%
$80,000 and above	5.0%

**Marital Status** Married/common law	44.4%
Single	35.8%
Previously married	19.8%

**RIASI—Total** mean (SD)	6.8 (4.8)

**RIASI—Recidivism** mean (SD)	3.3 (2.1)

**ADS** mean (SD)	1.8 (3.3)

**DAST** mean (SD)	0.3 (1.0)

**Previous DWI offence**	25.8%

Assigned to: Education program	73.8%
Treatment program	26.2%

**Table 2.** Association between RIASI factors and measures obtained in the assessment period. Back on Track remedial program clients (N = 22,298).

	**RIASI Factors** Regression coefficient (SE)^a^							

**Assessment Measures**	**Negative Affect**	**Sensation Seeking**	**Alcohol-Quantity**	**Social Conformity**	**High Risk Lifestyle**	**Alcohol Problems**	**Interpersonal Competence**	**Family History**
	
No. days of alcohol use	0.003*** (0.001)	n.s.	0.03*** (0.001)	0.001*** (0.0003)	002*** (0.0004)	−0.002*** (0.0004)	−0.002*** (0.0003)	n.s.
No. drinks per occasion	0.03*** (0.004)	0.05*** (0.002)	0.23*** (0.003)	0.01*** (0.002)	0.04*** (0.003)	−0.04*** (0.003)	−0.04*** (0.002)	−0.02*** (0.002)
ADS	0.18*** (0.003)	0.06*** (0.001)	0.19*** (0.003)	0.04*** (0.001)	0.07*** (0.002)	−0.22*** (0.002)	−0.07*** (0.002)	−0.05*** (0.001)
DAST	0.33*** (0.01)	0.18*** (0.004)	0.27*** (0.01)	0.05*** (0.004)	0.21*** (0.01)	−0.25*** (0.01)	−.13*** (0.01)	−0.07*** (0.004)
No. DWI charges	0.05*** (0.01)	n.s.	0.16*** (0.01)	0.04*** (0.005)	0.21*** (0.01)	−0.18*** (0.01)	−0.048*** (0.007)	−0.092*** (0.006)
No. DWI convictions	0.06*** (0.01)	n.s.	0.17*** (0.01)	0.05*** (0.006)	0.23*** (0.01)	−0.19*** (0.01)	−0.052*** (0.007)	−0.100*** (0.006)

Notes:

^a^ Regression coefficient and standard error (SE) values were rounded/truncated. Data controlled for age and gender. t-test significance

*** p < 0.005, n.s. = not significant.

**Table 3.** Association between RIASI factors and measures obtained in the follow-up period. Back on Track remedial program clients (N = 22,298).

	**RIASI Factors** Regression coefficient (SE)^a^							

**Follow-up Measures**	**Negative Affect**	**Sensation Seeking**	**Alcohol-Quantity**	**Social Conformity**	**High Risk Lifestyle**	**Alcohol Problems**	**Interpersonal Competence**	**Family History**
	
No. days of alcohol use	−0.002*** (0.001)	n.s.	0.02*** (0.001)	0.002*** (0.0003)	n.s.	0.002*** (0.001)	n.s.	n.s.
No. drinks per occasion	n.s.	0.04*** (0.002)	0.17*** (0.004)	0.01*** (0.002)	0.03 *** (0.003)	n.s.	−0.018*** (0.002)	−0.017*** (0.002)
Health Problems	0.55*** (0.08)	0.13*** (0.04)	0.45*** (0.08)	0.12*** (0.03)	n.s.	−0.41*** (0.06)	−0.132*** (0.04)	−0.194*** (0.036)
Blackout or memory problems	1.68*** (0.14)	0.56*** (0.07)	1.34*** (0.13)	n.s.	0.37*** (0.10)	−1.60*** (0.10)	−0.601*** (0.077)	−0.392*** (0.064)
Mood changes	0.66*** (0.08)	0.27*** (0.04)	0.94*** (0.08)	0.15*** (0.03)	0.32*** (0.06)	−0.59*** (0.06)	−0.295*** (0.045)	−0.190*** (0.037)
Problem in relationship	0.81*** (0.10)	0.27*** (0.05)	0.74*** (0.10)	0.13*** (0.04)	0.43*** (0.08)	−0.68*** (0.08)	−0.37*** (0.057)	n.s.
Problem controlling aggression	1.83*** (0.16)	0.33*** (0.08)	1.35*** (0.16)	n.s.	0.63*** (0.12)	−1.40*** (0.12)	−0.71*** (0.090)	n.s.
School/work problems	0.06*** (0.01)	−0.01*** (0.004)	n.s.	n.s.	n.s.	−0.03*** (0.01)	n.s.	n.s.
Legal Problems	0.53*** (0.12)	0.25*** (0.06)	0.47*** (0.11)	n.s.	n.s.	−0.43*** (0.09)	−0.214*** (0.064)	−0.155*** (0.054)
Financial problems	0.96*** (0.11)	0.30*** (0.05)	0.91*** (0.11)	n.s.	0.53*** (0.08)	−0.77*** (0.08)	−0.440*** (0.062)	−0.172*** (0.052)

Notes:

^a^ Regression coefficient and standard error (SE) values were rounded/truncated. Data controlled for age and gender. t-test significance

*** p < 0.005, n.s. = not significant.

**Table 4.** Association between the RIASI factors and use of health services (no. sessions/visits/meetings attended) in the follow-up period. Back on Track remedial program clients (N = 22,298).

	**RIASI Factors** Regression coefficient (SE)^a^							

**Follow-up Measures**	**Negative Affect**	**Sensation Seeking**	**Alcohol-Quantity**	**Social Conformity**	**High Risk Lifestyle**	**Alcohol Problems**	**Interpersonal Competence**	**Family History**
	
Assessment/Outpatient Drug/Alcohol treatment facility	0.06*** (0.005)	n.s.	0.03*** (0.01)	0.01*** (0.002)	n.s.	−0.07*** (0.004)	n.s.	−0.02*** (0.002)
Mental Health facility—outpatient	0.07*** (0.01)	n.s.	n.s.	0.02*** (0.004)	n.s.	−0.05*** (0.01)	n.s.	n.s.
Employee Assistance program	0.10*** (0.02)	n.s.	0.07*** (0.02)	n.s.	n.s.	−0.11*** (0.01)	n.s.	n.s.
Family/Marital Counseling	0.13*** (0.02) 0.06***	n.s.	n.s.	n.s.	n.s.	−0.07*** (0.02) −0.06***	n.s.	n.s.
Private doctor	(0.01)	n.s.	n.s.	n.s.	n.s.	(0.01)	n.s.	n.s.
Self-help—alcohol/drug problem	0.01*** (0.001)	n.s.	0.004*** (0.001)	0.003*** (0.0003)	0.004***(0.001)	−0.02*** (0.001)	−0.02*** (0.001)	−0.004*** (0.0003)
Self-help other—problem	n.s.	n.s.	n.s.	n.s.	n.s.	−0.02***(0.004)	n.s.	−0.01*** (0.002)

Notes:

^a^ Regression coefficient and standard error (SE) values were rounded/truncated. Data controlled for age and gender. t-test significance

*** p < 0.005, n.s. = not significant.

## **Appendix.** RIASI Factors and selected items (factor loadings > 0.26)

**Table:**

**Loading**	**Negative Affect** (eigenvalue 5.70)
0.41	I often feel so restless I can’t sit still.
0.51	I am irritated a great deal more than people are aware of.
0.41	I often feel like a powder keg ready to explode.
−0.30	I have no trouble sleeping or staying asleep.
0.27	I have experienced a major stressful life event in the past 12 months.
0.52	I have feelings that something bad will happen to me.
0.55	I feel like I have lost energy. I am fatigued and tired.
0.59	I often have feelings of nervousness.
0.64	I often feel sad or blue.
0.33	It depresses me that I did not do more for my parents.
0.56	I often feel hopeless about the future.
	**Sensation Seeking** (eigenvalue 2.17)
0.36	I sometimes do dangerous or risky things just for fun.
0.34	I often acted without thinking as a child.
0.51	In the past five years, how many jobs have you had?
0.30	If you go out drinking, how many places do you drink at in one evening?
	**Alcohol-Quantity** (eigenvalue 1.81)
0.40	After 7 or more drinks, I feel happier.
0.72	How much money do you usually spend on alcohol per week?
0.37	What is the largest number of drinks you ever consumed in a 24 hour period?
0.62	How many days of the week do you usually drink?
0.55	When you are drinking, how many drinks do you usually have?
0.52	How many drinks does it take before you begin to feel the effects of alcohol?
	**Social Conformity** (eigenvalue 1.40)
−0.29	When I don’t get my own way, I sulk or pout.
0.60	I am always courteous, even to people who are disagreeable.
−0.54	I sometimes feel resentful when I don’t get my way.
−0.61	No matter who I’m talking to, I’m always a good listener.
	**High Risk Lifestyle** (eigenvalue 1.35)
0.27	I have been arrested for crimes other than drinking and driving.
0.66	Since the age of 18, I have been accidentally cut, or cut in a fight, or burned enough to leave a scar.
0.68	Since the age of 18, I have needed emergency treatment for an injury of some kind.
0.32	I skipped school as a child.
0.38	How many traffic tickets for moving violations have you ever received (e.g., speeding, running a red light or a stop sign)?
	**Alcohol Problems** (eigenvalue 1.27)
−0.46	When I drink 7 or more drinks, I become aggressive.
−0.33	When the alcohol runs out, I leave a party.
−0.56	When I have a problem I try to make it go away by drinking.
0.35	I feel that I have lived the right kind of life.
−0.39	A drink or two gives me energy to get started.
−0.63	When I get beyond a certain point, I don’t stop drinking until all the booze is gone or I pass out.
0.48	I hardly ever drink more than I plan to.
−0.39	I was referred for a liver test, or a blood test for liver enzymes.
0.38	When I am drinking, I make sure I do not skip any meals.
	**Interpersonal Competence** (eigenvalue 1.20)
0.30	I have no problem telling a companion that he or she has done something to hurt my feelings.
0.29	When I make plans, I am almost certain to make them work.
0.28	I slow down when I traffic light turns yellow.
0.41	It is easy for me to turn down an unreasonable request for a friend.
0.33	I am probably not capable of slapping someone, even when I lose my temper.
0.50	I don’t like to break rules, even if I think they are wrong.
0.54	I am not interested in surprising or upsetting others by doing something that might shock them.
	**Family History** (eigenvalue 1.13)
−0.73	I have relatives who have had problems with alcohol or drugs.
−0.76	A family member was arrested for drinking and driving.
